# Congenital Fibrous Hamartoma of the Tip of the Tongue: A Benign Entity

**DOI:** 10.5826/dpc.1104a73

**Published:** 2021-10-01

**Authors:** Ambra Di Altobrando, Luca Casadio, Iria Neri

**Affiliations:** 1 AUSL della Romagna, Dermatology Unit, Ravenna, Italy; 2 Department of Pediatrics, Santa Maria delle Croci Hospital, Ravenna, Italy; 3 Department of Experimental, Diagnostic and Specialty Medicine, Dermatology, University of Bologna, Italy

## Case Presentation

**Figure 1:**
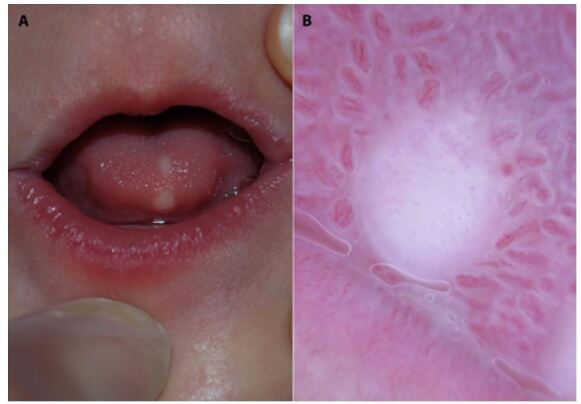
(A) Two dome-shaped pearly nodules on the dorsal surface of the tip of the tongue. (B) Dermoscopy showed subtle, radial, linear, and comma vessels over a pearly background.

A 1-month-old second-born girl was referred to our clinic due to congenital lesions affecting the tongue. The baby, born at full term, following an uncomplicated pregnancy, was otherwise healthy and breastfeeding, and her family members reported no medical history of note. Clinical examination showed 2 dome-shaped pearly nodules of 0.1 cm in maximum diameter on the dorsal aspect of the tip of her tongue (Figure 1A). Dermoscopy revealed subtle, radial, linear, and comma vessels over a pearly background (Figure 1B). Diagnosis of congenital fibrous hamartomas of the tip of the tongue was made.

## Teaching Point

Congenital fibrous hamartoma of the tip of the tongue consists of 1 or 2 asymptomatic pearly or yellowish nodules, not exceeding 0.5 cm in maximum diameter, located ventrally or dorsally at the tip of the tongue. It is not associated with cleft lip or palate, or with feeding problems. Dermoscopy, even if not diagnostic in this case, can be useful to highlight its benign features. Management includes regular follow-up, whereas surgical excision should be avoided, since the clinical picture is classic and stable over time [1].
